# Polymeric redox-responsive delivery systems bearing ammonium salts cross-linked via disulfides

**DOI:** 10.3762/bjoc.9.189

**Published:** 2013-08-13

**Authors:** Christian Dollendorf, Martin Hetzer, Helmut Ritter

**Affiliations:** 1 Lehrstuhl für Präparative Polymerchemie, Institut für Organische Chemie und Makromolekulare Chemie, Heinrich-Heine Universität, Universitätsstraße 1, Geb. 26.33.00, 40225 Düsseldorf, Germany

**Keywords:** cationic hydrogel, cross-linked polymer, 2-(dimethylamino)ethyl methacrylate (DMAEMA), disulfide cleavage, *N*,*N*-diethylacrylamide (DEAAm)

## Abstract

A redox-responsive polycationic system was synthesized via copolymerization of *N*,*N*-diethylacrylamide (DEAAm) and 2-(dimethylamino)ethyl methacrylate (DMAEMA). *N*,*N’*-bis(4-chlorobutanoyl)cystamine was used as disulfide-containing cross-linker to form networks by the quaternization of tertiary amine groups. The insoluble cationic hydrogels become soluble by reduction of disulfide to mercaptanes by use of dithiothreitol (DTT), tris(2-carboxyethyl)phosphine (TCEP) or cysteamine, respectively. The soluble polymeric system can be cross-linked again by using oxygen or hydrogen peroxide under basic conditions. The redox-responsive polymer networks can be used for molecular inclusion and controlled release. As an example, phenolphthalein, methylene blue and reactive orange 16 were included into the network. After treatment with DTT a release of the dye could be recognized. Physical properties of the cross-linked materials, e.g., glass transition temperature (*T*_g_), swelling behavior and cloud points (*T*_c_) were investigated. Redox-responsive behavior was further analyzed by rheological measurements.

## Introduction

Hydrogels with stimuli-responsive properties, in combination with targeted release of embedded drugs, have emerged as a fascinating class of biomedical materials and attracted great interest in medical science, polymeric biotherapeutics and drug delivery in general [1–4]. In this regard, poly(*N*-isopropylacrylamide) (PNIPAM) and poly(*N*,*N*-dimethylamino)ethyl methacrylate (PDMAEMA) based hydrogels are the most extensively studied representatives. Due to the protonation of covalently attached tertiary amine groups, PDMAEMA exhibits pH-responsive behavior and is used in gene delivery [5–7]. In general, temperature- and pH-responsive hydrogels containing hydrophilic monomers show low toxicity along with a high efficiency in drug delivery [8]. Besides, cross-linked cationic nanogels have been suggested as DNA delivery systems [9–11]. Hamamoto et al. reported a cross-linked polycationic hydrogel when investigating the swelling–deswelling behavior and the absorbency of organic substances [12]. Positively charged polymeric systems show an increased adsorption to negatively charged cell membranes, a higher retention time within tumor tissue [13–15] and the ability of DNA encapsulation used in anti-cancer treatment [16–23]. Further examples for polymeric networks based on poly(ethylenimine) (PEI) or poly(diethyl acrylamide) (PDEA) can be found in the literature [24–26].

Disulfide bonds are generally stable in human blood circulation and extracellular milieus, but are prone to cleavage in a reductive environment through dithiol–disulfide exchange reactions forming two corresponding thiol end-group bearing moieties [27–28]. The cleavage reaction is relatively fast and can take place within minutes to hours. This is considerably faster than the kinetics of other functionalities like esters and carbonates, whose degradation take days to weeks or even months inside the human body [29–31]. Dithiothreitol (DTT), tris(2-carboxyethyl)phosphine (TCEP) and glutathione tripeptide (γ-glutamyl-cysteinyl-glycine; GSH) are known as the most common disulfide-cleaving agents [32–33]. Furthermore, GSH is the most abundant biological reductive agent in the human body with a 50–1000 fold excess in human cells compared to extracellular milieus. In tumor cells the GSH concentration is even higher [34–35]. This large difference can be exploited for selective intracellular delivery and release of bioactive agents like DNA or low molecular-weight drugs [34]. Especially in the human blood stream the molecular weight is of significant importance for prolonged circulation cycles and enhanced selectivity for diffusion into tumor cells (enhanced permeation–retention effect) [13,36–41].

Reductive cleavable polymeric networks can release embedded drugs within the reductive tumor-cell environment [34,42]. There are several examples for micellar polymer systems, which bear disulfide functionalities and lose their structure after a cleavage induced by the addition of DTT, and which subsequently release the embedded substances, such as dyes or DNA molecules [33,43–45]. Other examples for disulfide cross-linked hydrogel networks are copolymers of *N*-(2-hydroxypropyl)methacrylamide (HPMA) and *N*-methacryloylglycylglycine, copolymers of DMAEMA and 1,4-bis(2-thiobenzoylthio)prop-2-yl)benzene, or cross-linked poly(L-lysine) [46–48].

In the current work, we describe the synthesis of a novel redox-responsive polycationic hydrogel by cross-linking copolymers of *N*,*N*-diethylacrylamide (DEAAm) and DMAEMA with a disulfide-bearing cross-linking agent. The redox-responsive behavior of the cross-linked poly(DEAAm-*co*-DMAEMA) hydrogel was shown by reversible enclosure and release of different dyes and further proven by rheological measurements. Additionally, physical properties of the cationic hydrogels, e.g., glass transition temperature (*T*_g_), swelling behavior, and cloud points (*T*_c_) of the thiol-bearing copolymers after cleavage were investigated. Although our results show only preliminary investigations, this hydrogel system with its redox-responsive behavior and polycationic structure could be a promising system for medical applications.

## Results and Discussion

### Synthesis of cross-linked hydrogels in solution and inclusion-release investigations of embedded dyes

The copolymerization of DEAAm and DMAEMA was carried out in ethyl acetate as solvent using different concentrations (25–50 wt %) and monomer ratios (1:1 and 5:1). Quantitative conversions were reached. Solutions with higher concentrations led to higher molecular weights during the reaction, but also to broader weight distributions (refer to Table 1).

**Table 1:** Results for the copolymerization of DEAAm and DMAEMA using different monomer ratios.

DEAAm/DMAEMA	conc. [wt %]	AIBN [mol %]	temp. [°C]	time [h]	conversion [%]	*M*_n_,_GPC_ [g mol^−1^]	PDI

1:1	25	0.5	60	18	>99	37200	3.20
5:1	25	0.5	60	18	>99	71500	2.72
1:1	50	0.5	60	18	>99	57100	4.47
5:1	50	0.5	60	18	>99	121400	3.27
1:1	bulk	0.25	varying^a^	varying^a^	>99	187400	4.30

^a^16 h at 60 °C, 8 h at 70 °C, 16 h at 80 °C, and 3 h at 100 °C.

**Scheme 1:**
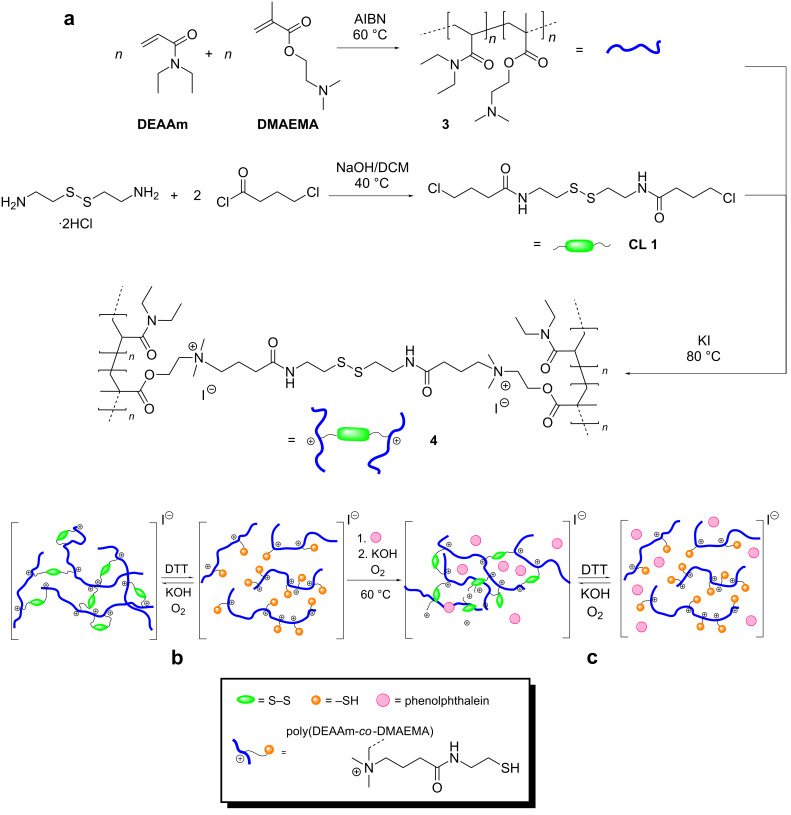
(a) Synthesis of a polycationic cross-linked hydrogel containing disulfide groups. (b) Reductive cleavage and oxidative rebuilding of disulfide groups in the polymeric network using DTT and KOH/O_2_. (c) Enclosure and release of embedded dyes by reductive opening and oxidative rebuilding of the polymeric network.

The cross-linking agent *N*,*N’*-bis(4-chlorobutanoyl)cystamine (**CL 1**) was synthesized by using the reaction of cystamine dihydrochloride and 4-chlorobutanoyl chloride [49]. Polycationic hydrogels where obtained by adding **CL 1** to a solution of poly(DEAAm-*co*-DMAEMA) and potassium iodide in acetone at 80 °C. The polymeric network was formed via quaternization of the tertiary amine groups of DMAEMA with both halogen atoms of **CL 1** (Scheme 1a). Potassium iodide was added to accelerate the quaternization by replacing the halogen atoms in a Finkelstein reaction. Using poly(DEAAm-*co*-DMAEMA) with a molar ratio of 1:1 (sample **3**) resulted in a better network formation, compared to a molar ratio of 5:1, because more tertiary amine groups are available for the halogen atoms of **CL 1** to form cationic networks via quaternization reactions. Additionally, the use of a DMAEMA/**CL 1** ratio of 2:1 results in a higher possibility of each tertiary amine groups to react with halogen atoms to form quaternary ammonium groups. A high polymer concentration in the reaction mixture should lead to a higher network-density based on increased intermolecular cross-linking in contrast to intramolecular quaternization reactions in diluted solution. After cross-linking of the copolymers by quaternization, the insoluble polycationic hydrogels were filtered off and washed several times with acetone to remove unreacted **CL 1** and potassium iodide.

**Figure 1:**
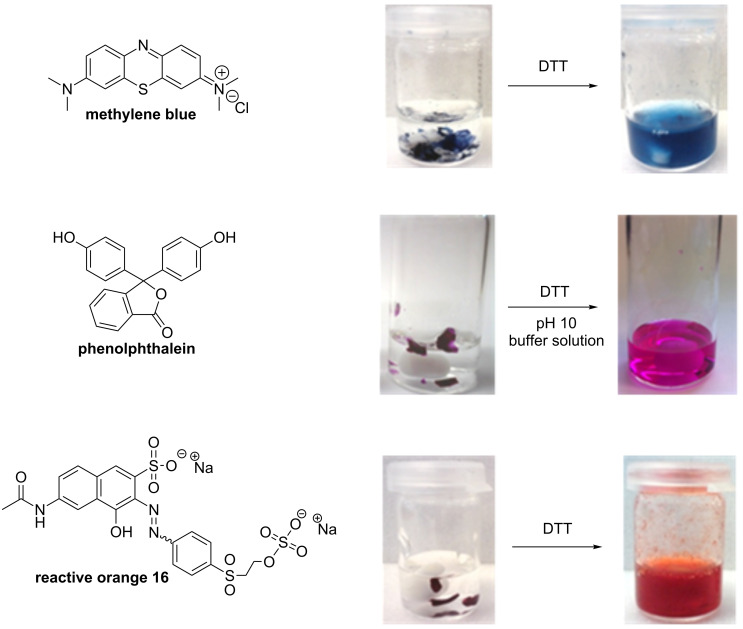
Release of enclosed dyes from polycationic networks containing disulfide bonds after treatment with DTT.

To determine the redox-responsive behavior of the cross-linked polymers, they were given into water with a subsequent addition of DTT to dissolve the polymeric networks by the reduction of the disulfide bonds to thiol side-chains (Scheme 1b). The dissolved polymers were cross-linked again by the oxidation of the thiol groups to reform disulfide bonds using oxygen under basic conditions at 60 °C. As described above, it was crucial to use concentrated solutions, because only thiol groups of different polymeric chains in close proximity can form cross-linked disulfide bonds and, therefore, polymeric networks. As an example for the enclosure of drugs or other molecules, the dyes phenolphthalein, methylene blue and reactive orange 16, respectively, were embedded into the polycationic networks. Therefore, the dyes were simply added to a solution of linear polymers containing thiol groups and an oxidizing agent (Scheme 1c). After filtering-off the obtained cross-linked polymers and several washing steps, the samples showed no solubility and no diffusion of the embedded dyes in water after 24 hours, which was indicating a stable inclusion. As can be seen in Figure 1, only upon addition of DTT at room temperature the polymeric networks were cleaved again and the dyes were released.

Since for the above described experiments an excess of DTT in relation to **CL 1** was used, it should be noted that DTT cannot only reduce disulfide bonds to thiols but it can also reduce the functional groups of dyes or substances. For example, methylene blue showed an initial loss of color during the release investigations due to the reduction by DTT, but regained it after exposure to oxygen as the leuco form of methylene blue was oxidized again.

### Synthesis of cross-linked polymer discs in bulk

**Figure 2:**
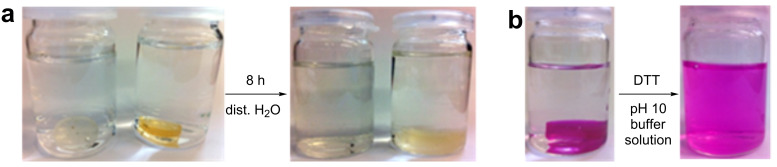
(a) Behavior of poly(DEAAm-*co*-DMAEMA) (left) and cross-linked poly(DEAAm-*co*-DMAEMA) (right) in distilled water. (b) Reductive cleavage of cross-linked poly(DEAAm-*co*-DMAEMA) networks containing phenolphthalein in pH 10 buffer solution.

To investigate the physical properties of polycationic hydrogels, polymer discs were synthesized in bulk polymerizations using a polytetrafluoroethylene (PTFE) plate containing several cavities (refer to Supporting Information File 1). In this way, the polymerization of the monomers DEAAm and DMAEMA, along with cross-linking using **CL 1**, are combined to obtain cross-linked polymer discs in a one-step reaction. Because of the higher concentration, polymerizations in bulk without cross-linking agents lead to higher molecular weights (Table 1). These factors enable better network formations and more efficient intermolecular cross-linking reactions in bulk. In order to avoid the saturation of tertiary amine groups during quaternization reactions, the concentrations of the used cross-linking agents were chosen to be between 1.0 and 5.0 mol % in relation to the combined amount of monomers DEAAm and DMAEMA. Figure 2a shows the different solubility of poly(DEAAm-*co*-DMAEMA) discs (sample **3**) compared to poly(DEAAm-*co*-DMAEMA) discs cross-linked with **CL 1** (samples **4**) in distilled water. While the soluble hydrogel dissolved completely after 8 h, the cross-linked polycationic hydrogel networks remained insoluble even after days.

The enclosure of dyes could also be accomplished in a one-step reaction along with polymerization and quaternization to give colored polycationic discs. When phenolphthalein was used, the polymer discs showed a characteristic violet coloration upon immersion into pH 10 buffer solution. Depending on the used concentration of cross-linker, a slight release of dye molecules from the discs outer shell could be recognized after an initial swelling of the polymer discs. However after removing the supernatant and adding fresh water, the coloration of the polymer disc remained constant and no further diffusion was observed. This indicates that solvent molecules can diffuse into the network structure, but the dye molecules remain enclosed. Treatment with DTT lead to the expected structural loss and dissolving of the polymer discs, along with the release of the embedded phenolphthalein molecules (refer to Figure 2b).

**Scheme 2:**
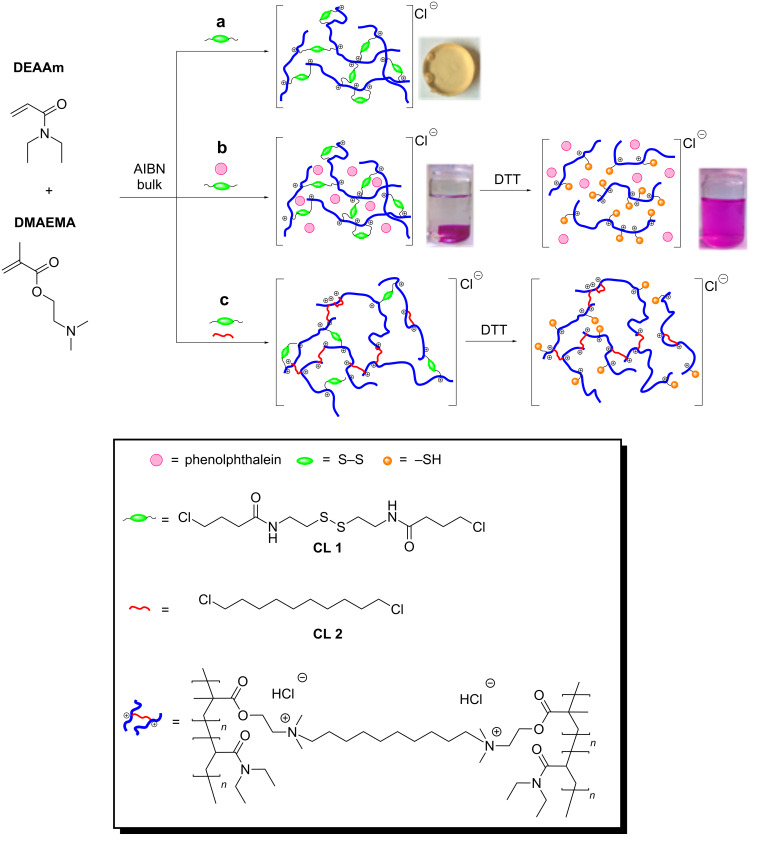
Synthesis of poly(DEAAm-*co*-DMAEMA) discs. (a) Cross-linked discs with **CL 1**. (b) Cross-linked discs with **CL 1** and phenolphthalein. (c) Cross-linked discs with **CL 1** and **CL 2**.

To determine the effect of the reduction of the disulfide bonds along with the structural loss of cross-linked polycationic networks, 1,10-dichlorodecane (**CL 2**) was used as a non-reducible cross-linker. It ensures form-stability of the polymer discs containing both cross-linking agents **CL 1** and **CL 2**. In this way, the reduction of disulfide bonds in polymer discs could be investigated with swelling experiments and rheological measurements comparing polymer discs containing either only **CL 1** or **CL 2**, or both cross-linkers (Scheme 2).

### Rheological measurements of cross-linked polymer discs

**Figure 3:**
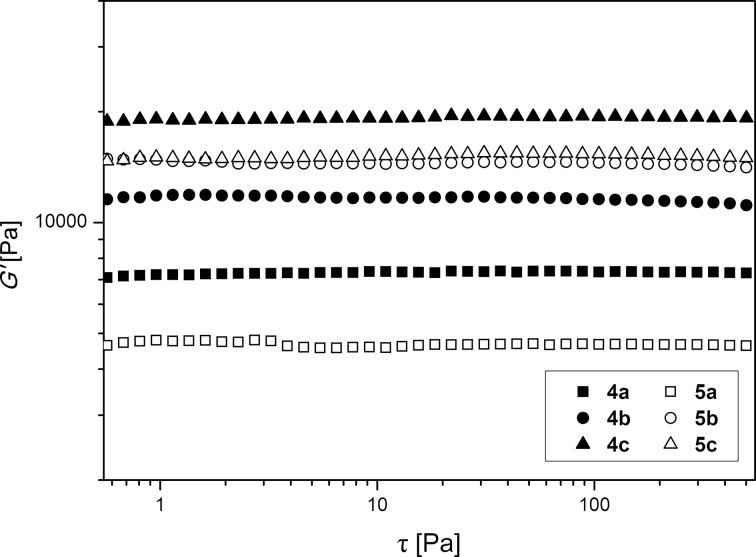
Storage modulus G’ as function of applied shear stress τ for samples **4** and **5** containing different amounts of **CL 1** or **CL 2**.

To investigate the rheological characteristics of cross-linked polycationic hydrogels, polymer discs of poly(DEAAm-*co*-DMAEMA, 1:1) containing between 1.0 and 5.0 mol % of either **CL 1** (samples **4**), or **CL 2** (samples **5**), or both cross-linkers (samples **6**) were synthesized. The samples were immersed in distilled water until the equilibrium state of swelling was reached. The swollen samples were then used for oscillatory measurements using the serrated plate–plate geometry. As expected, the storage modulus G’ showed significant higher values than the loss modulus G’’ for all samples over the whole range of applied shear stresses. This corresponds with the behavior of a cross-linked gel. When comparing the storage modulus G’ the polymer discs showed a general increase of rigidity with increasing amounts of cross-linker. For samples **4**, G’ increases from 7300 Pa for 1.0 mol % (**4a**), to 11600 Pa for 2.5 mol % (**4b**), and to 18900 Pa for 5.0 mol % (**4c**). A similar trend could be seen for samples **5**, where G’ increases from 4600 Pa for 1.0 mol % (**5a**), to 14500 Pa for 2.5 mol % (**5b**), and to 15300 for 5.0 mol % (**5c**) (Figure 3 and Table 2). The polymer discs containing **CL 1** show slightly higher values for G’ and therefore a higher rigidity of the polymer network when containing 1.0 and 5.0 mol % cross-linker. This behavior can be explained by the fact that the structure of **CL 1** includes two amide functionalities that enables the formation of intermolecular hydrogen bonds. Interestingly, the samples containing 2.5 mol % cross-linker show an opposite behavior. Here **5b** containing **CL 2** exceeds the G’ value of **4b** containing **CL 1,** which cannot be clearly explained.

**Table 2:** Results for oscillatory measurements of polymer discs containing **CL 1** and / or **CL 2** after immersion in different solvents.

sample	CL 1 [mol %]	CL 2 [mol %]	solvent	G’ [Pa] ^a^	σG’ [Pa] ^a^

**4a**	1.0	-	dist. water	7300	340
**4b**	2.5	-	dist. water	11600	407
**4c**	5.0	-	dist. water	18900	303
**5a**	-	1.0	dist. water	4600	66
**5b**	-	2.5	dist. water	14500	245
**5c**	-	5.0	dist. water	15300	123
**6a**	5.0	1.0	dist. water	24700	1234
	5.0	1.0	0.03 M DTT	9000	160
**6b**	5.0	2.5	dist. water	38500	1578
	5.0	2.5	0.03 M DTT	22000	291
**6c**	5.0	5.0	dist. water	56900	1867
	5.0	5.0	0.06 M DTT	44500	872
	5.0	5.0	30% H_2_O_2_	49400	1685

^a^The presented values are the average results of three measurements.

**Figure 4:**
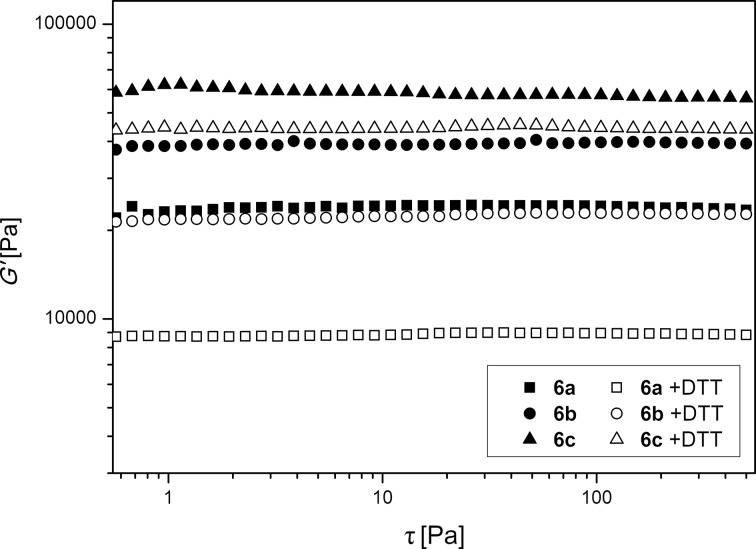
Storage modulus G’ of samples **6** immersed in water (full symbols) or DTT solution (empty symbols) containing 5.0 mol% **CL 1** and different amounts of **CL 2**.

To demonstrate the redox-responsive character of **CL 1** and to prove the reversibility of cleavage and rebuilding of the disulfide functionality, polymer discs containing a fixed amount of 5.0 mol % **CL 1** and various amounts of **CL 2** were synthesized (samples **6**). The amount of **CL 2** was chosen as 1.0 mol % (**6a**), 2.5 mol % (**6b**) or 5.0 mol % (**6c**) to ensure form stability of the polymer discs even after the disulfide functionalities were cleaved. The polymer discs were immersed into distilled water and rheological measurements were conducted as described above. As expected, all samples showed the behavior of cross-linked gels and the values of the storage modulus G’ increased from 24700 Pa for **6a**, to 38500 Pa for **6b**, and to 56900 Pa for **6c**. All values were higher than for polymer discs containing only either **CL 1** or **CL 2**. The samples were then immersed in aqueous DTT solutions for three days. In order to maximize the cleavage of the disulfide functionalities in the polycationic network DTT, concentrations of 0.03 M and 0.06 M were chosen depending on the amount of **CL 1**. As can be seen in Figure 4, after immersion in the DTT solution all samples showed decreased G’ values indicating less rigid network structures (refer to Table 2).

The decrease of G’ ranged from 15700 Pa for **6a** to 22300 Pa for **6c** and can be attributed to the cleavage of the disulfide functionalities into free thiol groups. Since the values of samples **6** after cleavage of **CL 1** with DTT are still higher than G’ measured for samples **5** containing only **CL 2**, it is most likely that not all disulfide functionalities were cleaved or that other interactions, possibly of the thiol-bearing side chains, are involved. Nevertheless, the decrease of the G’ values shown for all samples proves the redox-responsive character of **CL 1**.

**Figure 5:**
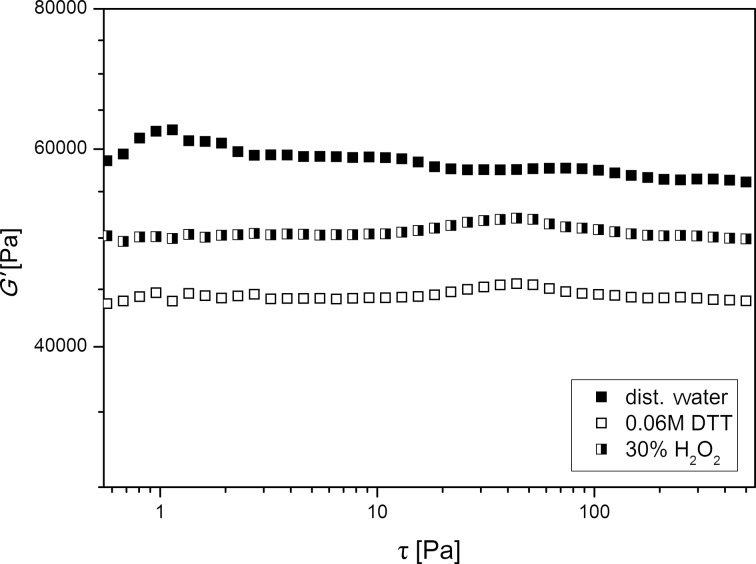
Storage modulus G’ of a polymer disc **6c** containing 5.0 mol % **CL 1** and 5.0 mol % **CL 2** after being immersed in distilled water (full symbols), 0.06 M DTT solution (empty symbols) and 30% H_2_O_2_ (half symbols).

The reversibility of cleavage and rebuilding of the cross-linking disulfide functionalities was investigated by immersion of **6c** (5 mol % **CL 1** and 5 mol % **CL 2**) in aqueous 30% H_2_O_2_. In a first attempt, a treatment with KOH and oxygen at 60 °C, after swelling and reduction of the disulfide bonds, the polymer discs lost their stability and were partially destroyed. Therefore, a gentler oxidation method was used by immersion of the polymer discs in aqueous 30% H_2_O_2_ at room temperature. After three days the sample was subjected to oscillatory measurements again and the value for G’ was compared to the values received after immersion in distilled water and aqueous DTT solution. Although G’ did not reach its initial value of 56900 Pa, there is an increase of 4900 Pa to 49400 Pa in comparison to 44500 Pa after the disulfide cleavage in DTT (refer to Figure 5 and Table 2). This is a clear sign that only a partial rebuilding of the disulfide linkages takes place. One explanation for the discrepancy to the initial value could be side reactions of excess DTT with cleaved thiol groups and therefore less cross-linkage within the network structure. Another explanation for this observation could be the further oxidation of thiol groups to sulfonic acid by H_2_O_2_.

### Investigations of material properties *Q*, *T*_g_, and *T*_c_

**Figure 6:**
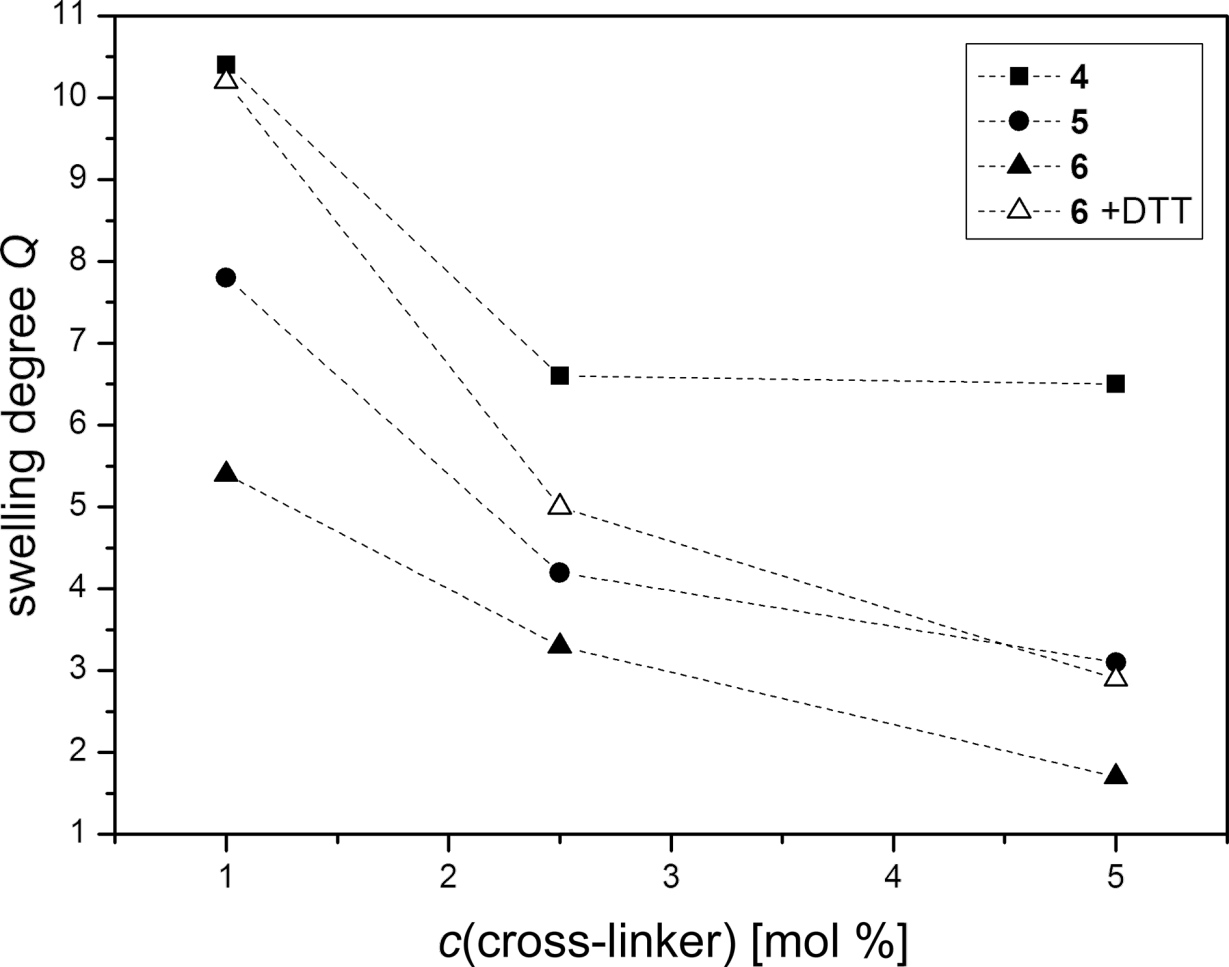
Swelling degree *Q* of polymer discs **4–6** as a function of the amount and type of containing cross-linker.

To investigate the swelling behavior *Q* of the polymer discs samples **4** to **6** were immersed into 10 mL of distilled water until the equilibrium degree of swelling and a constant weight were achieved, which usually took up to three days. The swelling degree *Q* was determined using the following formula: *Q* = [*m*_swollen_ − *m*_dry_]/*m*_dry_, where *m*_swollen_ is the mass of the swollen polymer disc and *m*_dry_ is the mass of the dried sample. As can be seen in Figure 6, sample **4** showed the highest swelling degrees of all samples.

Compared to sample **5**, the swelling degrees of sample **4** are higher by about 2.5 to 3.5, depending on the amount of cross-linker used (Table 3). This can be explained by the structure of **CL 1** which contains two amide groups and one disulfide functionality. Because of this polymer networks containing **CL 1** can form more hydrogen bonds in contrast to networks containing **CL 2**, and the enhanced affinity for interactions with water molecules leads to a better enclosure of water and therefore a higher swelling degree. Sample **6**, which contains both cross-linkers, shows the lowest values for *Q*. This can be attributed to its overall higher content of cross-linker, compared to samples **4** and **5**. However, after immersion of **6a** and **6b** in 0.03 M DTT solution, and of **6c** in 0.06 M DTT solution, and the subsequent cleavage of the disulfide functionalities, the swelling degrees increase considerably for all samples. The swelling degree for **6a** increases from 5.41 to 10.15, for **6b** from 3.25 to 5.0 and for **6c** from 1.69 to 2.92. The values for **6a** and **6b** are even higher than the swelling degrees of the samples containing only **CL 2**. This enhanced swelling behavior can be explained by the additional thiol-bearing side chains after the cleavage of the disulfide functionalities. The side chains lead to an enlargement of the polymer structure and an increased interaction with water molecules. The influence of the side chains decreases with increasing amounts of **CL 2** resulting in nearly identical values of *Q* for **5c** and **6c**.

**Table 3:** Results for the determination of swelling degree *Q* and glass transition temperature *T*_g_ for samples **3–6**.

sample	*m*_dry_	*m*_swollen_ (water)^a^ [g]	m_swollen_ (DTT)^a^ [g]	*Q*	*Q*_DTT_	*T*_g_^b^ [°C]

**3**	—	—	—	—	—	43
**4a**	0.38	4.33	—	10.39	—	47
**4b**	0.38	2.89	—	6.61	—	51
**4c**	0.41	3.08	—	6.51	—	58
**5a**	0.31	2.74	—	7.84	—	47
**5b**	0.38	1.96	—	4.16	—	47
**5c**	0.37	1.52	—	3.11	—	49
**6a**	0.39	2.50	4.35	5.41	10.15	65
**6b**	0.40	1.70	2.40	3.25	5.00	65
**6c**	0.39	1.05	1.53	1.69	2.92	65

^a^Constant weights were achieved after 3 days of swelling. ^b^The presented values are the average results of three cycles.

The glass transition temperatures (*T*_g_) of the polymer networks were determined using differential scanning calorimetry (DSC). As a reference poly(DEAAm-*co*-DMAEMA, 1:1) (**3**) with a *T*_g_ of 43 °C was used. Cross-linking with **CL 2** slightly increases the *T*_g_ from 43 to 47 and 49 °C for **5a**, **5b** and **5c**, respectively. In contrast, samples containing **CL 1** showed a stronger influence of the amount of cross-linker used and *T*_g_ increased from 47 °C for **4a**, to 51 °C for **4b**, and up to 58 °C for **4c**. This behavior can be explained by additional intermolecular interactions of amide and disulfide functionalities introduced by **CL 1**. The higher the amount of **CL 1**, the more energy is needed to disrupt these interactions, which corresponds with an increasing *T*_g_. Interestingly, the combination of both cross-linkers yields glass transition temperatures of 65 °C for all samples, regardless of additional amount **CL 2** in samples **6**.

Polymers of DEAAm and/or DMAEMA are both known to show temperature-dependent solubilities with lower critical solution temperatures (LCST) of 32 and 50 °C, respectively, in neutral media [50–51]. Therefore, the temperature-dependent behavior of poly(DEAAm-*co*-DMAEMA) and the influence of thiol side-chains on the cloud point temperatures (*T*_c_) was investigated. Samples **3**, **4a**, **4b** and **4c** were dissolved in aqueous DTT solution under stirring until clear solutions were obtained. Then the polymer solutions were subjected to turbidity measurements. The concentration for all samples was chosen to be 10 mg·mL^−1^, and heating and cooling were carried out with a rate of 1 °C·min^−1^ in a temperature range from 15 to 70 °C. Sample **3** showed cloud points of 25.0 and 27.7 °C in distilled water and aqueous 0.03 M DTT solution, respectively. Depending on the amount of thiol side-chains, the temperature *T*_c_ of samples **4a**, **4b** and **4c** increases from 35.7, to 43.3, and up to 49.6 °C (refer to Table 4). This trend is similar to the increasing swelling degrees with increasing amounts of **CL 1**. The thiol side-chains increase the hydrophilicity by forming hydrogen bonds with the surrounding aqueous medium. Thus, more energy is needed to disrupt these interactions, leading to higher temperatures before cloud points are reached [42,52].

**Table 4:** Cloud point temperatures *T*_c_ of poly(DEAAm-*co*-DMAEMA) bearing thiole side chains after disulfide cleavage with DTT in dist. H_2_O and pH 10 buffer solution.

sample	solvent	CL 1 [mol %]	*T*_c_ (heating) [°C]	*T*_c_ (cooling) [°C]

**3**	dist. H_2_O	0.0	25.0	25.5
**3**	+0.03 M DTT	0.0	27.7	28.3
**4a**	+0.03 M DTT	1.0	35.7	36.3
**4b**	+0.03 M DTT	2.5	43.3	43.1
**4c**	+0.06 M DTT	5.0	49.6	48.6

**3**	pH 10 buffer solution	0.0	22.6	22.8
**3**	+0.03 M DTT	0.0	23.7	23.8
**4a**	+0.03 M DTT	1.0	23.0	22.5
**4b**	+0.03 M DTT	2.5	25.6	23.3
**4c**	+0.06 M DTT	5.0	27.9–31.0	26.9

Since poly(DMAEMA) is not only a temperature-responsive, but also a pH-responsive polymer, the same measurements were conducted in pH 10 buffer solution. To ensure a good comparability the polymer concentration was held constant at 10 mg·mL^−1^ and the same heating and cooling rate of 1 °C·min^−1^ was used. Sample **3** showed cloud points of 22.6 in pH 10 buffer solution and 23.7 °C with 0.03 M DTT. This is more than 10 °C lower than the LCST of poly(DMAEMA) in pH 10 buffer solution reported in the literature [50]. The cloud points for samples **4a** and **4b** just differed slightly with temperatures *T*_c_ of 23.0 and 25.6 °C and only **4c** showed an elevated cloud point in the range of 27.9 to 31.0 °C. Under basic conditions the thiol side-chains are deprotonated, leading to betaine structures. Thus, the negatively charged thiolate groups can interact with the cationic ammonium salts, either intramolecular in the same polymer or intermolecular with other polymer chains. This results in additional electrostatic interactions between the polymer chains leading to decreased cloud points.

## Conclusion

Novel redox-responsive polycationic hydrogels of DEAAm and DMAEMA were successfully synthesized by cross-linking reactions via quaternary ammonium compounds with the disulfide-bearing cross-linker **CL 1**. The ability of the polymer network to enclose and release substances by reductive cleavage or oxidative formation of disulfide bonds was shown exemplarily using different dyes. The redox-responsive character was proven by oscillatory rheological measurements and different material properties of polycationic polymer discs were investigated. Because of its polycationic structure, this polymeric system could be a promising compound for complexation of DNA-like substances. Its ability for selective release in reductive environments like tumor tissues could possibly be used in medical applications or chemotherapy.

## Supporting Information

Supporting information features experimental procedures, descriptions of characterization methods, copies of NMR spectra and Figures of heating/cooling cycles of turbidity measurements.

File 1 Experimental section and characterization methods.
