# Supplementary material for: Polymeric redox-responsive delivery systems bearing ammonium salts cross-linked via disulfides
Source: Beilstein J Org Chem. 2013 Aug 13;9:1652–62. doi: 10.3762/bjoc.9.189 (PMC3778402; doi:10.3762/bjoc.9.189)
Supplement: File 1 — Experimental section and characterization methods. [file Beilstein_J_Org_Chem-09-1652-s001.pdf]

Supporting Information

for

Polymeric redox-responsive delivery systems bearing ammonium salts cross-linked via disulfides

Christian Dollendorf[†], Martin Hetzer[†] and Helmut Ritter*

Address: Lehrstuhl für Präparative Polymerchemie, Institut für Organische Chemie und Makromolekulare Chemie, Heinrich-Heine Universität, Universitätsstraße 1, Geb. 26.33.00, 40225 Düsseldorf, Germany

[†]Both authors contributed equally to this work.

Email: Helmut Ritter - h.ritter@uni-duesseldorf.de

*Corresponding author

Experimental section and characterization methods

Materials

1,10-Dichlorodecane (Sigma-Aldrich, 99%), 4-chlorobutanoyl chloride (Fluka, 98%), cystaminedihydrochloride (Acros, 96%), cysteamine (Wako Chemicals, 98%), dithiothreitol (DTT; Acros, 99%), hydrogen peroxide solution (AppliChem, 30% (w/w) in water), magnesium sulfate (Grüssing, 99%), methylene blue hydrate (Acros, 96%), phenolphthalein (Roth, 99%), potassium hydroxide (Sigma-Aldrich, 85%), potassium iodide (Acros , 99%), reactive orange 16 (Sigma-Aldrich, 50% dye content), sulfuric acid (Sigma Aldrich, 95%), sodium chloride (VWR, 99.7%), sodium hydroxide (AppliChem, 99%), sodium hydrogencarbonate ($NaHCO_3$; Grüssing, 99.5%), tris(2-carboxyethyl)phosphine hydrochloride (TCEP; Sigma-Aldrich, 98%), and buffer solution pH 10.00 ± 0.02 (Roth) were used as received. 2,2'-Azobis(2-methylpropionitrile) (AIBN; Fluka, 99%) was recrystallized twice from methanol. Acetone, dichloromethane (DCM), ethyl acetate, methanol and *n*-hexane (all technical grade) were distilled and stored over molecular sieve prior to use. *N*,*N*-diethylacrylamide (DEAAm; TCI, 99%) and 2-(Dimethylamino)ethyl methacrylate (DMAEMA; Sigma-Aldrich, 98%) were passed over a short column of basic alumina (Acros, 50–200 μm) prior to use.

Synthesis of *N,N'*-bis(4-chlorobutanoyl)cystamine (CL 1).

According to the literature procedure [1], in a 2000 mL Schlenk flask cystamine dihydrochloride (17.0 g, 75.49 mmol, 1.0 equiv) was dissolved in 2 M NaOH (500 mL) and DCM (250 mL). Under slow stirring 4-chlorobutanoyl chloride (25 mL, 223.40 mmol, 2.95 equiv) in DCM (100 mL) was added dropwise. After stirring for 16 h at 40 °C, the organic phase was washed with 1 M sulphuric acid (250 mL), 1 M $NaHCO_3$ (250 mL) and brine (125 mL). The organic phase was dried over magnesium sulfate, filtered and concentrated in vacuo. The solid residue was recrystallized from *n*-hexane/acetone (10:7) and dried in vacuo to give the product as a white crystalline solid (16.95 g, 46.91 mmol, 62%).

^{1}H NMR (600 MHz, $CDCl_3$): δ 6.50 (s, 2H, N***H***), 3.57 (m, 8H, 2x C***H***$_2$-Cl + 2x C***H***$_2$-NH), 2.80 (t, ^3J = 6.49 Hz, 4H, 2x C***H***$_2$-S), 2.38 (t, ^3J = 7.18 Hz, 4H, 2x C***H***$_2$-C=O), 2.09 (m, 4H, 2x CH_2-C***H***$_2$-CH_2) ppm; ^{13}C NMR (150 MHz, $CDCl_3$): δ 173.32 (2x ***C***=O), 44.73 (2x ***C***H$_2$-Cl), 38.63 (2x ***C***H$_2$-NH), 37.89 (2x ***C***H$_2$-S), 33.24 (2x ***C***H$_2$-C=O), 28.22 (2x CH_2-***C***H$_2$-CH_2) ppm; ESI (acetone) *m*/*z*: [M] calcd for $C_{12}H_{22}Cl_2N_2O_2S_2$, 361.35; found, 361.

Exemplary procedure for the copolymerization of DEAAm and DMAEMA in solution using different monomer ratios

In a 25 mL round-bottom-flask DEAAm (500 mg, 3.93 mmol, 1.0 equiv), DMAEMA (618 mg, 3.93 mmol, 1.0 equiv) and AIBN (6.46 mg, 0.039 mmol) were dissolved in ethyl acetate (1.25 mL) to reach a monomer concentration of 50 wt %. The solution was purged with nitrogen for 2 h before the reaction vessel was placed in an oil bath at 60 °C. After 18 h the polymerization was stopped by freezing the now solidified reaction mixture in liquid nitrogen. A NMR-sample was taken for the determination of conversion and $CDCl_3$ was added. A conversion of 99% was calculated based on the NMR data (see the Characterization Methods section for details of the calculation). The polymer was diluted in ethyl acetate, precipitated in *n*-hexane, filtered off, washed with *n*-hexane several times and dried in vacuo to give poly(DEAAm-*co*-DMAEMA, 1:1) as a white solid (1.08 g, 98%, GPC(DMAc): $M_{n,GPC}$ = 57100 g·mol^{-1}, PDI = 4.47).

^{1}H NMR (600 MHz, $CDCl_3$): δ 4.37-3.84 (2H, O-C***H***$_2$), 3.73-2.98 (4H, 2x N-C***H***$_2$), 2.79-2.47 (3H, backbone C***H*** +CH_2-C***H***$_2$-N), 2.43-2.21 (6H, 2x N-C***H***$_3$), 2.14-0.79 (13H, 2x backbone C***H***$_2$ + 2x N-CH_2-C***H***$_3$ + C-C***H***$_3$) ppm;

Exemplary procedure for the cross-linking of poly(DEAAm-*co*-DMAEMA) with CL 1 in solution

Poly(DEAAm-*co*-DMAEMA) (1:1, $M_{n,GPC}$= 57100 g·mol^{-1}; 950 mg, 3.36 mmol, 1.0 equiv) and **CL1** (606 mg, 1.67 mmol, 0.5 equiv) were dissolved in acetone (20 mL). Potassium iodide (500 mg, 3.01 mmol, 2.0 equiv) was added and the reaction mixture was stirred for 8 h at 80 °C under reflux. The solid residue was precipitated in diethyl ether, filtrated and dried in vacuo to give the insoluble cross-linked product as a white solid (1.41 g, 91%).

Exemplary procedure for the copolymerization of DEAAm and DMAEMA and cross-linking in bulk

In a 25 mL round-bottom-flask DEAAm (4.0 g, 31.5 mmol, 1.0 equiv), DMAEMA (4.9 g, 31.41 mmol, 1.0 equiv) and **CL 1** (1.23 g, 3.4 mmol, 0.1 equiv, 5.0 mol %) were stirred at 40 °C. The reaction mixture was purged with nitrogen for 1 h, before AIBN (26.0 mg, 0.158 mmol, 0.005 equiv) was added. The clear solution was transferred into a polytetrafluoroethylene (PTFE) disc containing several cavities of 1 cm diameter and 1 mm depth. The cavities were filled with the reaction mixture, covered with a polyoxymethylene (POM) foil and compressed with a screwed poly(methyl methacrylate) (PMMA) panel. The PTFE disc was stored in a cabinet dryer for 16 h at 60 °C, for 8 h at 70 °C, for 16 h at 80 °C and finally for 3 h at 100 °C. After cooling to room temperature, the polymer discs were taken from the cavities of the PTFE disc.

For the polymer discs with permanent cross-linking 1,10-dichlorodecane (**CL 2**) was used instead of **CL1** and for the polymer discs with both cross-linkers **CL1** and **CL2** were mixed. The same procedure was employed independent from the cross-linkers used.

Exemplary procedure for the cleavage of disulfide bonds in polycationic networks of poly(DEAAm-*co*-DMAEMA)

CL 1 cross-linked poly(DEAAm-*co*-DMAEMA) (1:1, 0.25 g) was stirred in distilled water (4 mL) together with DTT (50 mg, 0.14 mmol) for 24 h at room temperature until the insoluble network was completely dissolved.

Cross-linked polymer discs of poly(DEAAm-*co*-DMAEMA, 1:1) with 1.0 and 2.5 mol % **CL 1** were inserted in 0.03M DTT solution and polymer discs with 5.0 mol % **CL 1** in 0.06 M DTT solution. The polymer concentration was 10 mg·mL^{-1} and all discs were immersed in solution for three days before turbidity or rheological measurements.

Characterization methods

Nuclear magnetic resonance (NMR) measurements were conducted on a BrukerAvance III 600 spectrometer at 600 MHz for hydrogen nuclei and 150 MHz for carbon nuclei for structure verification. For the determination of the conversion of the copolymerizations of DEAAm and DMAEMA, the integrals of one vinylic proton (6.26–6.34 ppm) and the integrals of the side chain methylene protons of the polymers (2.80–3.80 ppm) were employed.

Size exclusion chromatography (SEC) with *N*,*N*-dimethylacetamide (DMAc) as eluent containing 0.03 wt % LiBr was performed on a Polymer Laboratories PL-GPC 50 Plus Integrated System, comprising an autosampler, a PLgel 5 μm bead-size guard column (50 × 7.5 mm) followed by three PLgel 5 μm MixedC columns (300 × 7.5 mm) and a differential refractive index detector at 50 °C with a flow rate of 1.0 mL·min^{-1}. The SEC system was calibrated against linear poly(styrene) standards or poly(methyl methacrylate) standards with molecular weights ranging from 160 to 6 × 10^6 g·mol^{-1}. All SEC calculations for poly(DMAAm) were carried out relative to a poly(styrene) calibration.

Rheological measurements were carried out using a Thermo Scientific HAAKE Mars II rheometer equipped with a serrated plate-plate geometry (plate diameter = 35 mm) and a temperature control system DC30/K10 from Thermo scientific to ensure constant temperatures with deviations of ±0.5 °C. Oscillatory shear measurements were performed for all polymer discs to achieve amplitude sweeps. All measurements were carried out at 20 °C and a constant normal force between both plates of 10 N. Controlled shear stress tests were recorded at a constant frequency of 1 Hz and shear stresses varying from 0.1 to 500 Pa in cycles of three measurements with 300 s recovery time between each measurement.

Electrospray ionization mass spectrometry (ESI-MS) was performed on an Ion-Trap-API Mass Spectrometer Finnigan LCQ Deca from Thermo Quest. The instrument was calibrated in the *m*/*z* range up to 2000 Da and the samples were dissolved in acetone.

Turbidity measurements were performed in a turbidity photometer from Tepper GmbH. The measurements were conducted using a voltage controlled semiconductor laser and a silicium photodiode (λ = 590 nm). For the preparation of the samples, dried polymer discs were dissolved using 0.03M DTT solutions (1.0 and 2.5 mol % **CL 1**) or 0.06 M DTT solutions (5.0 mol % **CL1**) of distilled water and buffer solution pH 10.00, respectively. The polymer concentration for all solutions was 10 $mg \cdot mL^{-1}$ and the heating was carried out with a rate of 1 $°C \cdot min^{-1}$ in a temperature range of 15 to 70 °C in distilled water and 15 to 35 °C in buffer solution pH 10.00.

Differential scanning calorimetry (DSC) measurements were performed using a Mettler Toledo DSC 822 controller apparatus in a temperature range between 0 and 190 °C with a heating rate of 15 $°C \cdot min^{-1}$. The values for the glass transition temperature point (T_g) reported are taken from the average of third heating cycles.

For the determination of the swelling degree the polymer discs were immersed into 10 mL of distilled water for 24 h. After being taken from the swelling medium excess water was removed from the surfaces and the discs were weighed. This procedure was repeated until the

equilibrium degree of swelling and a constant weight was achieved. The swelling degree Q was determined using the following formula: $Q = [m(\text{swollen}) - m(\text{dry})]/m(\text{dry})$.

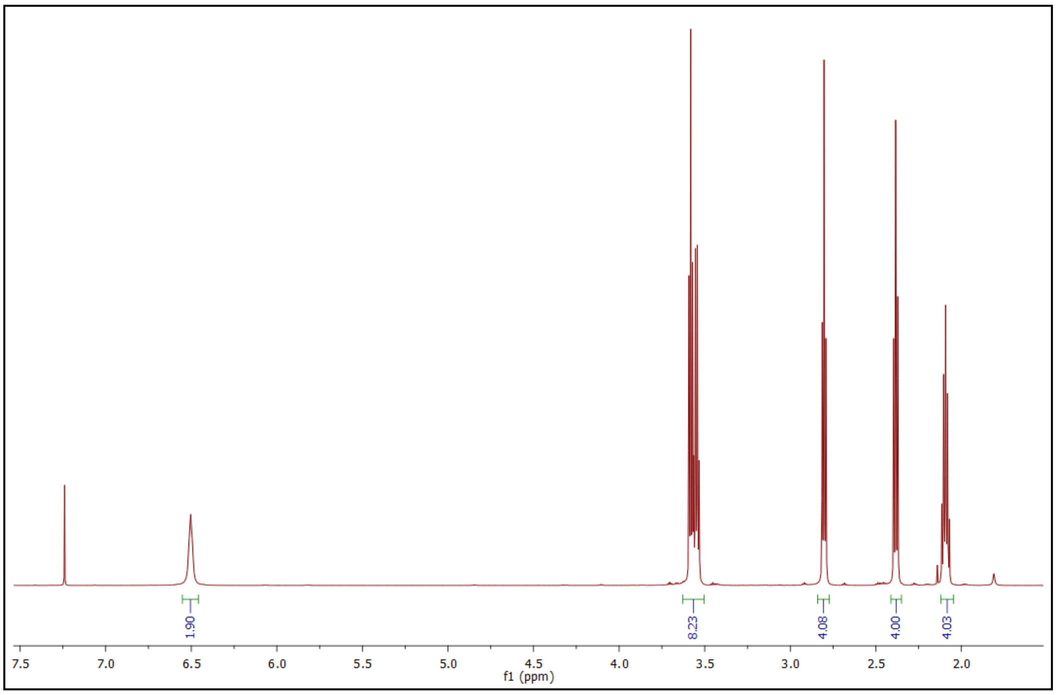

Figure S1: ^{1}H NMR spectrum of *N*,*N'*-bis(4-chlorobutanoyl)cystamine (**CL 1**).

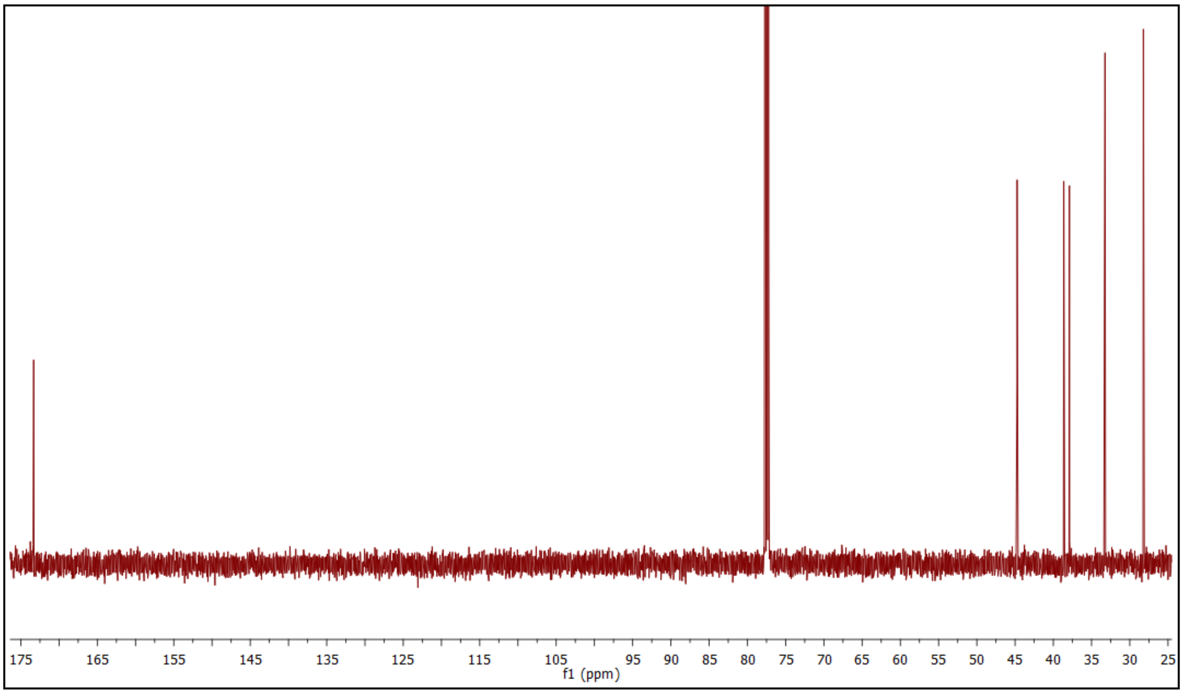

Figure S2: ^{13}C NMR spectrum of *N*,*N'*-bis(4-chlorobutanoyl)cystamine (**CL 1**).

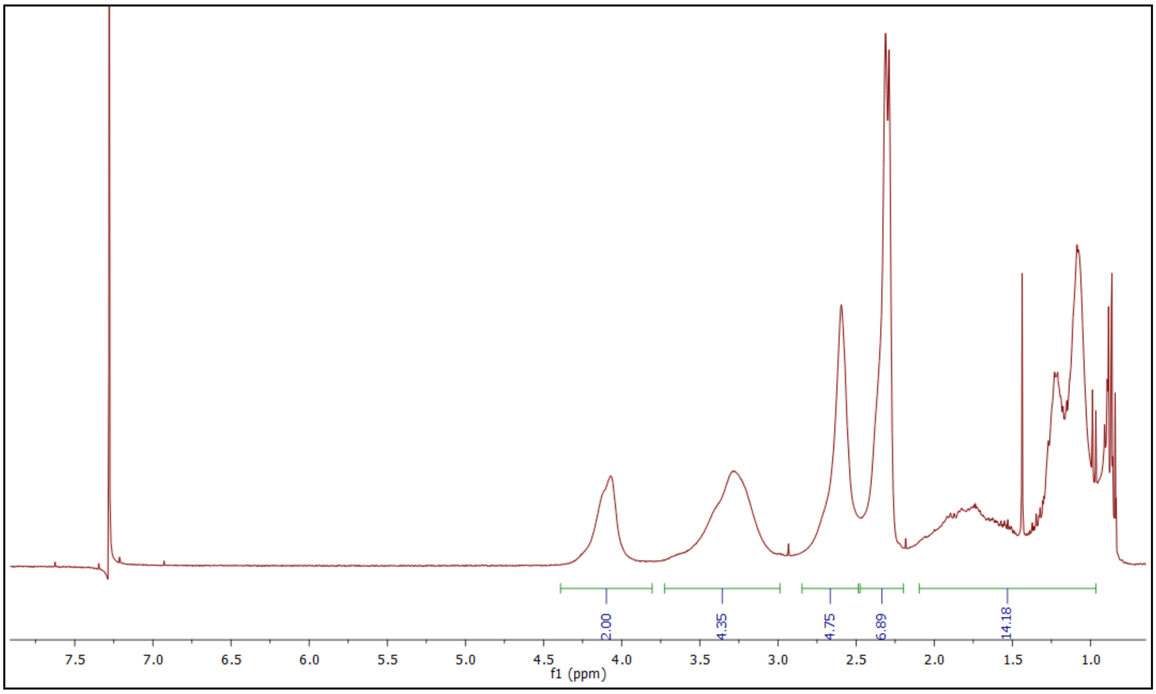

Figure S3: ^{1}H NMR spectrum of poly(DEAAm-*co*-DMAEMA) with a molar ratio of 1:1 (**3**).

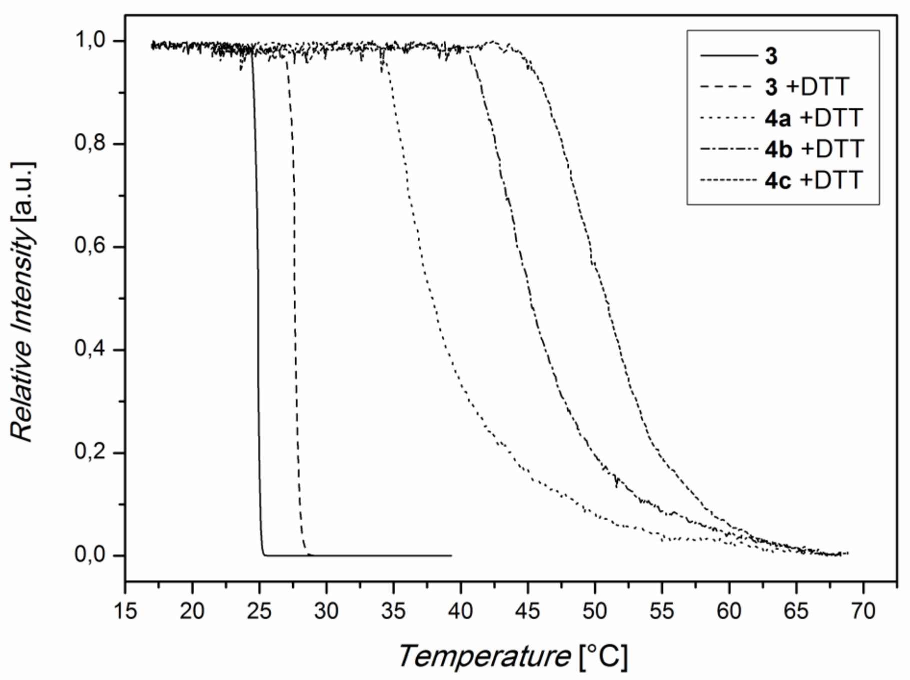

Figure S4: Heating cycles for turbidity measurements of samples **3** and **4** in distilled water.

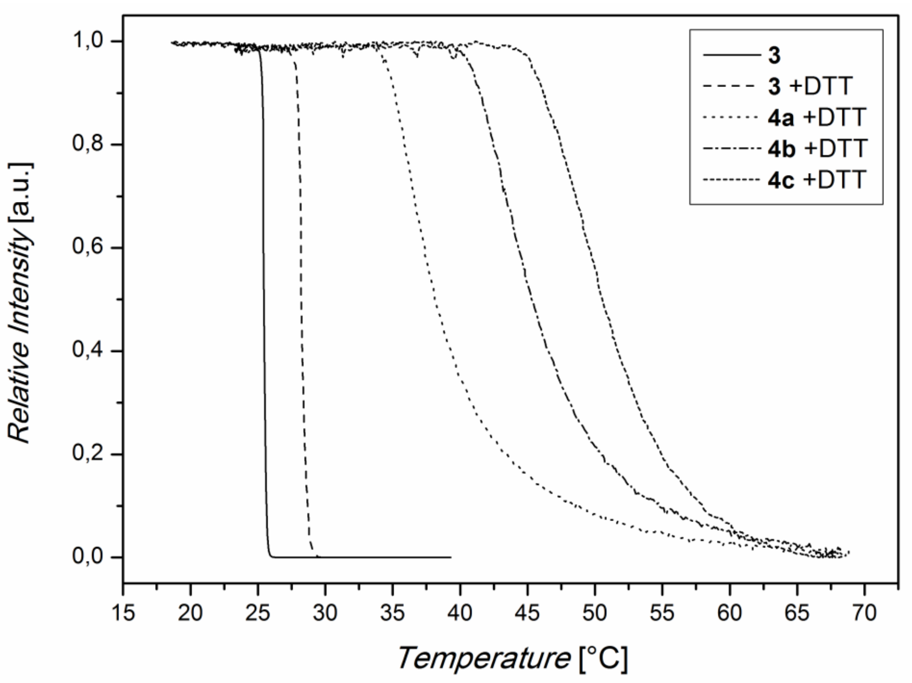

Figure S5: Cooling cycles for turbidity measurements of samples **3** and **4** in distilled water.

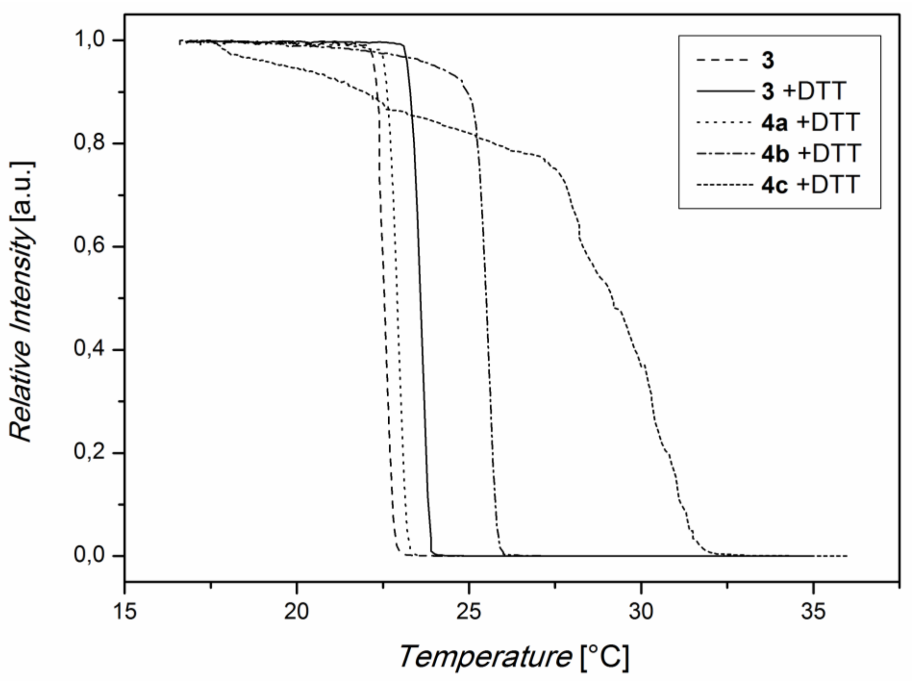

Figure S6: Heating cycles for turbidity measurements of samples **3** and **4** in pH 10 buffer solution.

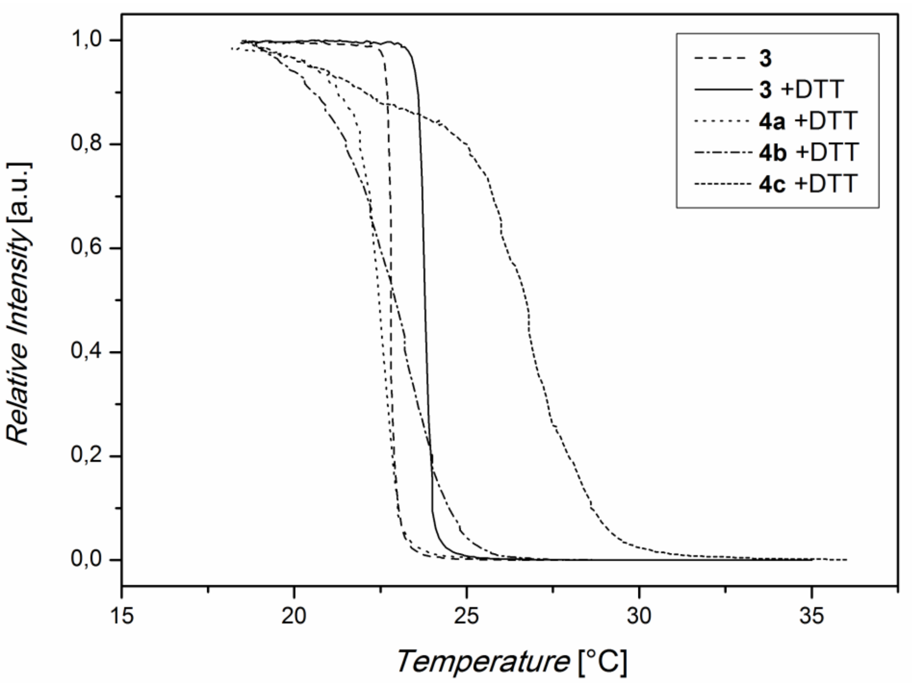

Figure S7: Cooling cycles for turbidity measurements of samples **3** and **4** in pH=10 buffer solution.

References

1. Bibart, R. T.; Vogel, K. W.; Drueckhammer, D. G. *J. Org. Chem.* **1999**, *64*, 2903-2909.
